# *Fusarium suttonianum* Identified as the Causal Agent of Root Rot in *Plukenetia volubilis* in Peru

**DOI:** 10.3390/jof11090642

**Published:** 2025-08-29

**Authors:** Elvin Delgado-Mera, Angel David Hernández-Amasifuen, Ángel Tuesta-Casique, Julio Santiago Chumacero-Acosta, Gerry Antonio Cosme-Garate, Gladstone Alves da Silva, Fernando Marcelo Carvajal Vallejos, Ronan Xavier Corrêa, Mike Anderson Corazon-Guivin

**Affiliations:** 1 Laboratorio de Biología y Genética Molecular, Universidad Nacional de San Martín, Jr. Amorarca N° 315, Morales 22201, Peru; edmera.ppgv@uesc.br (E.D.-M.); adhernandezama@gmail.com (A.D.H.-A.); atuesta@unsm.edu.pe (Á.T.-C.); juliochumacero90@gmail.com (J.S.C.-A.); gerry270485@gmail.com (G.A.C.-G.); 2 Center of Biotechnology and Genetics, Department of Biological Sciences, Universidade Estadual de Santa Cruz, Rodovia Jorge Amado Km 16, Ilheus 45662-900, Brazil; ronanxc@uesc.br; 3 Departamento de Micologia, Centro de Biociências, Universidade Federal de Pernambuco, Av. da Engenharia s/n, Recife 50740-600, Brazil; gladstone.asilva@ufpe.br; 4 Unidad de Limnología y Recursos Acuáticos (ULRA), Department of Biology, Faculty of Science and Technology (FCyT), University Mayor de San Simón, Calle Sucre Frente al Parque la Torre s/n, Zona las Cuadras, Cochabamba 00591, Bolivia; fmcvalle@yahoo.com

**Keywords:** root rot, *Fusarium suttonianum*, pathogenicity, agricultural plantation

## Abstract

Sacha inchi (*Plukenetia volubilis*) is a high-value crop due to its high content of omega-3 fatty acids and its outstanding nutritional, pharmaceutical, and cosmetic properties. However, this species faces challenges from diseases, particularly root rot. In this study, we identified one of the causal agents of root rot in sacha inchi using morphological observations, molecular methods, and pathogenicity tests. The pathogen was isolated from root tissues showing symptoms of *Fusarium* infection, observed in a plot in the Picota province. Morphological identification, DNA sequencing, and phylogenetic analysis using the *ITS* and *TEF-1α* markers revealed that the isolate causing root rot was *Fusarium suttonianum* (FSSC 20). Analysis of the PQ636870 (*ITS*) and PQ639345 (*TEF-1α*) sequences in the NCBI database, together with phylogenetic analysis, revealed 99.58% and 99.51% similarity with the *ITS* and *TEF* sequences, respectively, corresponding to *F. suttonianum*. Pathogenicity tests confirmed that this species induced the same symptoms observed in the field, fulfilling Koch’s postulates. This study represents the first report of *F. suttonianum* as a pathogen causing root rot in sacha inchi in Peru. This finding is critical for developing effective strategies for disease management and control, contributing to the sustainability and improvement of sacha inchi production in the region.

## 1. Introduction

*Plukenetia volubilis* L., commonly known as sacha inchi, is a perennial climbing plant belonging to the Euphorbiaceae family. It is native to the Amazon basin, particularly the Peruvian rainforest and northwestern Brazil [1,2,3,4,5]. Considering its remarkable nutritional and health properties, this species is extensively cultivated across Central and South America, as well as in several Asian countries [2,4,6,7,8].

The seeds of *Plukenetia volubilis* are composed of 22–30% protein and are an exceptional source of essential polyunsaturated fatty acids, including 35.2–50.8% α-linolenic acid (ω-3) and 33.4–41.0% linoleic acid (ω-6), both critical for human health [1,2,3,5,6,9]. Additionally, the seeds exhibit antioxidant properties and contain small amounts of monounsaturated fatty acids, such as oleic acid (ω-9), alongside saturated fatty acids like palmitic and stearic acids [2,6,9,10,11,12]. These qualities have established *P. volubilis* as a commercially significant crop in the food, pharmaceutical, and cosmetic industries [8,10,13].

In the San Martin region, Peru, *P. volubilis* is primarily cultivated as a monoculture, with smaller proportions grown alongside forestry species or annual crops in a variety of soil types [2]. According to Manco [14], since the establishment of the first field plantations, this species has exhibited susceptibility to *Fusarium* spp., with symptoms already observable at the seedling stage. From the second year of cultivation onwards, and throughout the production phase, this pathogen, often in association with *Meloidogyne* spp., causes considerable economic losses in the region. Root rot is regarded as one of the most significant diseases affecting *P. volubilis*, with the potential to cause yield losses of up to 100%, thereby contributing to the low productivity reported in the San Martin region [15]. Nevertheless, despite the economic importance of this fungal pathogen, current knowledge of root rot diseases in sacha inchi remains limited. In particular, no studies have documented its etiology through molecular identification techniques, and there is a lack of scientifically validated methods for the prevention and control of these diseases. In Peru, the presence of *Fusarium* species has recently been reported in other crops, such as pitahaya (*Selenicereus megalanthus*) [16], oregano (*Origanum vulgare*) [17] and banana (*Musa* spp.) [18]. However, to date, there are no official first reports of root rot caused by *Fusarium* spp. in *P. volubilis* in Peru.

The San Martín region is the leading producer, contributing 64.5% of the country’s total production, in 2023, the cultivation of *P. volubilis* in the San Martín region covered 1803.00 hectares under production, with an average yield of 765.87 kg ha^−1^ [19]. However, despite its established cultivation, *P. volubilis* is still considered in a process of domestication [2,20]. A key challenge for this species, both in Peru and other cultivation regions, is its susceptibility to pests and diseases.

Significant threats include *Meloidogyne incognita* [21], bacterial wilt caused *by Ralstonia pseudosolanacearum* phylotype I [22], stem canker caused by *Macrophomina phaseolina*, *Colletotrichum siamense*, *Lasiodiplodia theobromae* and *L. pseudotheobromae* [23,24,25], and root rot caused by *Fusarium* species [26,27,28]. Pathogens such as *Fusarium solani*, *F. verticillioides*, and the *Meloidogyne*-*Fusarium* complex [29] are particularly notable. Among these, soilborne *Fusarium* stands out as a pathogen responsible for root and stem collar rot. It disrupts water and nutrient transport, limiting crop growth and productivity [26,27,30,31,32,33]. Globally, *Fusarium* is estimated to cause economic losses of 10–30% in various agricultural crops [31,34,35,36].

The *Fusarium* genus, first described by Link in 1809, is characterized by its canoe-shaped conidia [31,33,37,38,39]. It comprises numerous phytopathogenic species that affect a wide range of agriculturally important crops worldwide [40,41]. Additionally, *Fusarium* causes significant postharvest losses due to its production of mycotoxins, which reduce crop economic value and pose risks to human and animal health [42,43]. The widespread distribution of *Fusarium* is attributed to its high survival capacity under diverse and adverse climatic conditions [38,41,44].

Morphological characterization of *Fusarium* spp. has traditionally been the most common method for species identification, focusing on the shape and size of microconidia and macroconidia, chlamydospores, and colony characteristics such as mycelial pigmentation [38,45]. However, morphological identification can be complex and controversial, leading to debates among researchers. Despite these challenges, morphology remains a valuable tool, often complemented by molecular identification. Accurate identification of *Fusarium* spp. requires integrating morphological and molecular approaches [44,46,47].

In molecular taxonomy, the most commonly employed genes for the identification of Fusarium are the internal transcribed spacer (*ITS*) and the translation elongation factor 1-alpha (*TEF1-α*), owing to their high discriminatory power at the species level within the genus [48,49,50,51]. These markers have been widely validated and used in molecular identification studies of *Fusarium* spp. associated with various agricultural crops worldwide [52,53,54,55,56,57]. The combined use of *ITS* and *TEF1-α* provides robust taxonomic resolution for species delimitation in most cases; however, it is acknowledged that *ITS* alone may be insufficient to distinguish species within certain *Fusarium* species complexes [58].

Although other markers, such as β-tubulin (*TUB2*) and the subunits of RNA polymerase II (*RPB1* and *RPB2*), offer higher resolution and are recommended for multilocus sequence typing (MLST) studies aimed at detailed characterizations of genetic diversity [59], this study chose *ITS* and *TEF1-α* due to their extensive prior validation, availability of reference sequences, and proven effectiveness for initial identification in the specific context of the first report of *Fusarium suttonianum*. Nonetheless, the importance of incorporating multilocus markers in future studies to deepen the taxonomic and phylogenetic characterizations of isolates is recognized.

In this context. This study aimed to identify the causal agent of root rot in *P. volubilis* through morphological and molecular characterization and pathogenicity testing. We hypothesized that *F. suttonianum* is a previously unreported pathogen of sacha inchi in Peru.

## 2. Materials and Methods

### 2.1. Collection of Biological Samples

**Figure 1:**
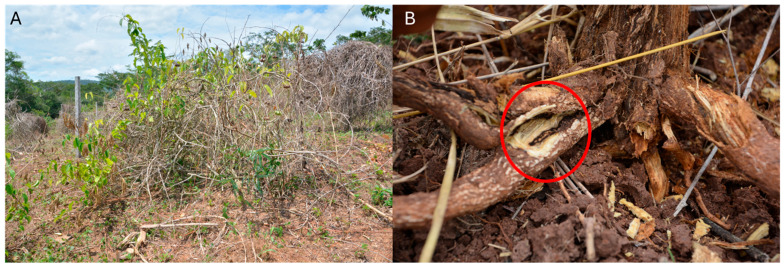
Root rot caused by sickle *Fusarium* in *P. volubilis* (**A**) Plant symptoms in the field; (**B**) Root symptoms.

Samples were collected from a symptomatic *Plukenetia volubilis* plant exhibiting typical disease characteristics, such as wilting, chlorosis, and stunting (Figure 1). Infected tissue samples were taken from the stem collar and root in an agricultural plantation located in the district of Leoncio Prado (6°59′21″ S, 76°13′32″ W, 255 m above sea level), province of Picota, San Martín department, Peru. The samples were placed in labeled plastic bags, stored in a cooler with ice, and transported to the laboratory for further analysis.

### 2.2. Isolation and Purification of the Pathogen

To isolate the pathogen, infected tissues from the diseased plant were first washed under running water to remove soil residues. The symptomatic tissues were then cut into small fragments of approximately 3 to 5 mm using a sterilized blade. These fragments underwent surface sterilization in 70% ethanol for 30 s and 1% sodium hypochlorite for 10 min. Subsequently, they were rinsed three times with sterilized water and dried using filter paper under a laminar flow hood. Within the same hood, sterilized fragments were transferred onto Petri dishes containing potato dextrose agar (PDA) (six pieces per plate) using sterile tweezers. Plates were incubated at 25 °C in darkness for three days, allowing fungal growth to emerge from the tissues. Pure cultures were obtained after three consecutive isolations using the hyphal-tip method [38,60,61].

### 2.3. Morphological Characterization

Morphological characteristics were analyzed from pure cultures incubated in three different media: PDA, Spezieller Nährstoffarmer Agar (SNA), and Carnation Leaf Agar (CLA). PDA cultures were used to observe colony pigmentation and aerial mycelium type. SNA cultures facilitated the formation of microconidia, while CLA was employed for homogeneous formation of macroconidia and chlamydospores [38,62,63].

Morphological descriptions were based on macroscopic (colony pigmentation and texture) and microscopic (microconidia, macroconidia, and chlamydospores) structures from 7-day-old cultures [64]. Images of these structures were recorded using a light microscope (NIKON, Eclipse E200, Tokyo, Japan) at 40× magnification, providing detailed visual documentation of the observed morphological features.

### 2.4. Molecular Characterization

Genomic DNA was extracted from 100 mg of mycelium obtained from 7-day-old PDA pure cultures using the modified cetyltrimethylammonium bromide (CTAB) method [29]. DNA concentration and quality were assessed using a spectrophotometer (NanoDrop™ One, Thermo Fisher Scientific, Waltham, MA, USA), and DNA integrity was evaluated by 1.0% agarose gel electrophoresis.

PCR amplification targeted the internal transcribed spacer (*ITS*) and the elongation factor 1-alpha (*TEF-1α*) genes. The *ITS* region was amplified using primers *ITS1* (5′-TCCGTAGGTGAACCTGCGG-3′) and *ITS4* (5′-TCCTCCGCTTATTGATATGC-3′) [65], while *TEF-1α* marker was amplified using primers *TEF1* (5′-ATGGGTAAGGA(A/G)GACAAGAC-3′) and *TEF2* (5′-GGA(G/A)GTACCAGT(G/C)ATCATGTT-3′) [66]. The final PCR reaction volume of 10 µL included 7.16 µL ultrapure water, 1 µL 10X reaction buffer, 0.2 µL 10 mM dNTPs, 0.4 µL 50 mM MgCl2, 0.2 µL of each primer (10 µM), 0.04 µL Platinum™ enzyme (5 U/µL), and 1 µL DNA at 50 ng/µL concentration.

For *TEF-1α*, PCR amplification was carried out under the following conditions: initial denaturation at 95 °C for 5 min; 40 cycles of 95 °C for 30 s, 54 °C for 45 s, and 72 °C for 2 min; followed by a final extension at 72 °C for 10 min, and cooling to 4 °C. For the universal *ITS* primer, amplification was performed under these conditions: initial denaturation at 95 °C for 5 min; 40 cycles of 95 °C for 40 s, 55 °C for 40 s, and 72 °C for 2 min; and a final extension at 72 °C for 10 min.

PCR products were detected via 1% agarose gel electrophoresis in 1X TAE buffer for 40 min and visualized using a gel documentation system (omniDOC Gel Documentation System, Cleaver Scientific, Rugby, UK). Amplified products were purified using the QIAEX II Gel Extraction Kit (QIAGEN, Venlo, The Netherlands) according to the manufacturer’s instructions and sent to MACROGEN for Sanger sequencing.

### 2.5. Phylogenetic Analysis

To reconstruct the phylogeny, an alignment (concatenate), based on *ITS* and *TEF-1α* gene, was generated with our nucleotide sequences and compared with *Fusarium* isolates available in GenBank (NCBI, http://www.ncbi.nlm.nih.gov/BLAST/, accessed on 14 April 2024). *F. oxysporum*, *F. incarnatum* and *F. equiseti* was included as an outgroup. Only sequences from isolates that presented data for these two genes were used in our study. The dataset was aligned in Mafft v.7 using the default parameters [67].

Prior to phylogenetic analyses, the model of nucleotide substitution was estimated using Topali 2.5 [68]. Bayesian (two runs over 1 × 10^6^ generations, with a sample frequency of 300 and a burning value of 25%) and maximum likelihood (1000 bootstrap) analyses were performed, respectively, in MrBayes 3.1.2 [69] and PhyML [70], launched from Topali 2.5, using the best model selected by the program (GTR + G). The DNA sequences corresponding to the *ITS* and *TEF-1α* loci of the *Fusarium suttonianum* isolate (code: LBGM-FUSA001) were deposited in the GenBank database.

### 2.6. Pathogenicity Assays

To fulfill Koch’s postulates and evaluate pathogenicity, the pure isolate LBGM-FF01, cultured for 7 days on PDA, was used to prepare the inoculum. The inoculum was prepared by culturing the fungus in potato dextrose broth (PDB), adding 5 × 5 mm agar plugs of fungal colonies into three 100 mL flasks containing PDB. These flasks were incubated at 25 °C for five days on a rotary shaker at 150 rpm to produce abundant conidia. The conidial suspension was filtered through three layers of sterile gauze, and its concentration was adjusted to 5 × 10^6^ conidia/mL using a hemocytometer.

For inoculation, pots were filled with 3 kg of a sterilized mixture of agricultural soil and sand (2:0.5, *v*/*v*). Pre-germinated sacha inchi seeds, surface-sterilized as described by Corazon-Guivin et al. [21], were sown. The isolate was inoculated onto 20-day-old seedlings by creating four equidistant holes (0.5 cm diameter, 4.0 cm depth) around each seedling. A small wound was made on the roots with a sterilized blade, and 20 mL of inoculum (1 × 10^8^ conidia per pot) was applied. For control plants, the roots were wounded and treated with sterile distilled water. Ten plants inoculated with the pathogen were evaluated, while another ten seedlings were used as controls. Plants were maintained in a greenhouse with daytime and nighttime temperatures ranging from 20 to 35 °C, enabling disease development and evaluation under conditions resembling natural environments. In *P. volubilis* plants, disease severity was assessed through visual observation, using an ordinal rating scale from 0 to 4, where 0 = no symptoms, 1 = yellowing, drying, and shedding of the first basal leaves, 2 = moderate leaf chlorosis progressing upwards, 3 = severe wilting and leaf defoliation progressing upwards, and 4 = plant death (complete defoliation and root rot).

## 3. Results

### 3.1. Morphological Characterization

**Figure 2:**
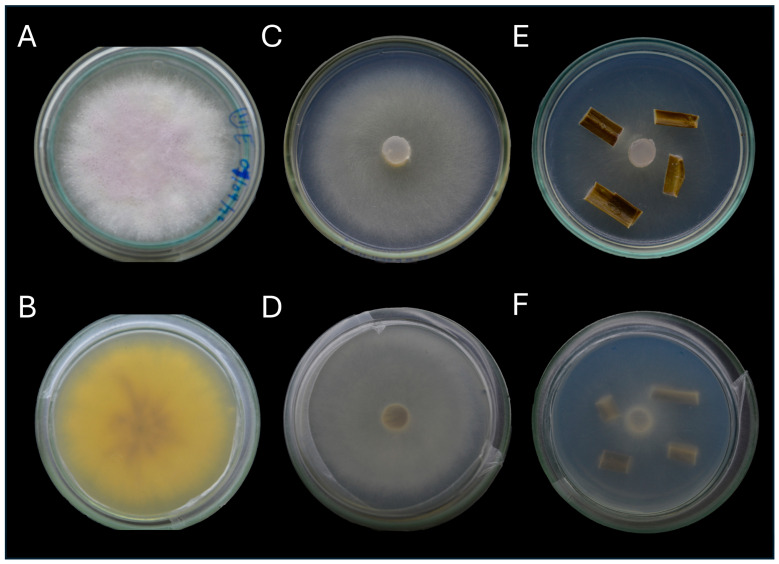
Macroscopic characteristics of *Fusarium suttonianum* in three culture media. (**A**,**B**) pigmentation of sickle *Fusarium* in PDA after seven days of incubation at 25 °C. Front and back of the colony. (**C**,**D**) pigmentation of sickle *Fusarium* in SNA after seven days of incubation at 25 °C. Front and back of the colony. (**E**,**F**). pigmentation of sickle *Fusarium* in CLA after seven days of incubation at 25 °C. Front and back of the colony.

The LBGM-FUSA001 strain was observed after seven days of incubation on different culture media. On PDA, the aerial mycelium appeared cottony-white on the front of the plate, turning yellowish on the reverse (Figure 2A,B). On SNA, the aerial mycelium was less cottony than on PDA, remaining white on both the front and reverse sides of the plate (Figure 2C,D). On CLA, the aerial mycelium was very sparse (Figure 2E,F).

**Figure 3:**
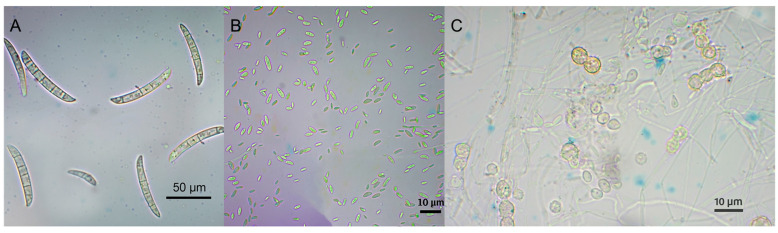
Micromorphological characteristics of *Fusarium suttonianum*. (**A**) macroconidia (Scale bar = 20 μm). (**B**) microconidia (Scale bar = 10 μm). (**C**) chlamydospores (Scale bar = 10 μm).

On PDA, the colony produced structures such as macroconidia, microconidia, chlamydospores, and phialides. The macroconidia exhibited 3–6 septa, were falcate (curved), with pointed apical cells and slightly hooked basal cells. Their dimensions ranged from 27.7 to 81.0 µm × 5.6 to 8.0 µm (*n* = 50) (Figure 3A). The microconidia, formed in false heads on monophialides, were hyaline, obovoid, ellipsoidal, and occasionally cylindrical, exhibiting a straight or curved shape with 0–2 septum. They measured 6.0 to 21.0 µm × 2.1 to 5.5 µm (*n* = 10) (Figure 3B). The phialides were subcylindrical and emerged from the conidiophores. The chlamydospores were abundant, spherical, intercalary or terminal, solitary or arranged in chains along the hyphae, and had rough walls. Their size ranged from 6.4 to 9.6 µm (*n* = 10) (Figure 3C).

### 3.2. Molecular Characterization and Phylogenetic Analysis

PCR amplification of the *ITS* and *TEF-1α* regions yielded fragments of 491 bp and 633 bp, respectively. The sequences of the LBGM-FUSA001 isolate were deposited in the NCBI GenBank under accession numbers PQ636870 (*ITS*) and PQ639345 (*TEF-1α*). BLASTn searches in GenBank identified the sequences as *Fusarium suttonianum* with a maximum identity of 99.58% and 99.51% to the NRRL 32858 strain with accession numbers DQ094617.1 (*ITS*) and DQ247163.1 (*TEF-1α*), respectively (Table 1).

A concatenated phylogenetic analysis of F. suttonianum was performed using ITS and TEF-1α sequences. The concatenated alignment had a length of 992 bp, with nucleotide frequencies of A = 0.250, C = 0.250, G = 0.250, and T = 0.250.

**Figure 4:**
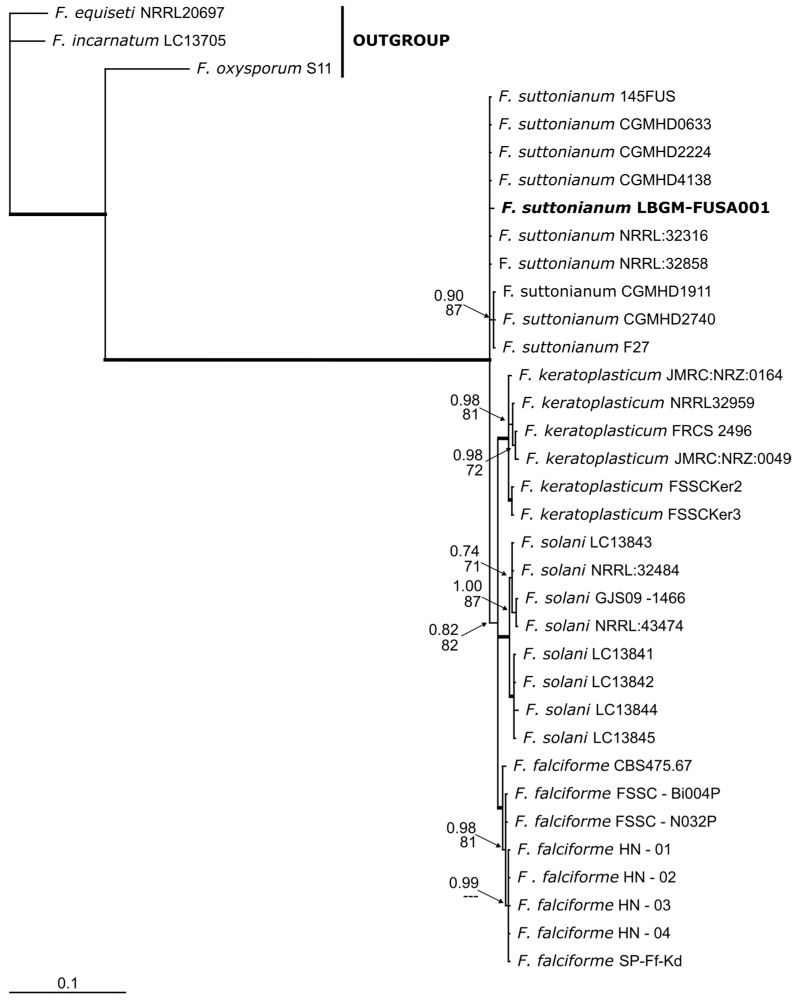
Phylogenetic tree obtained from the analysis of the *ITS* and *TEF-1α* genes. Sequences are labeled with their database accession numbers. Displayed, in that order, are Bayesian posterior probabilities (BI) ≥ 0.90 and maximum likelihood (ML) bootstrap values ≥ 70% based on 1000 replicates. Thick branches indicate clades with exceptional support in both analyses.

The resulting phylogenetic trees showed bootstrap support values > 90%, grouping our sequences in the same clade with other *F. suttonianum* isolates, providing strong evidence for the taxonomic placement of the isolate (Figure 4).

### 3.3. Pathogenicity Assays

**Figure 5:**
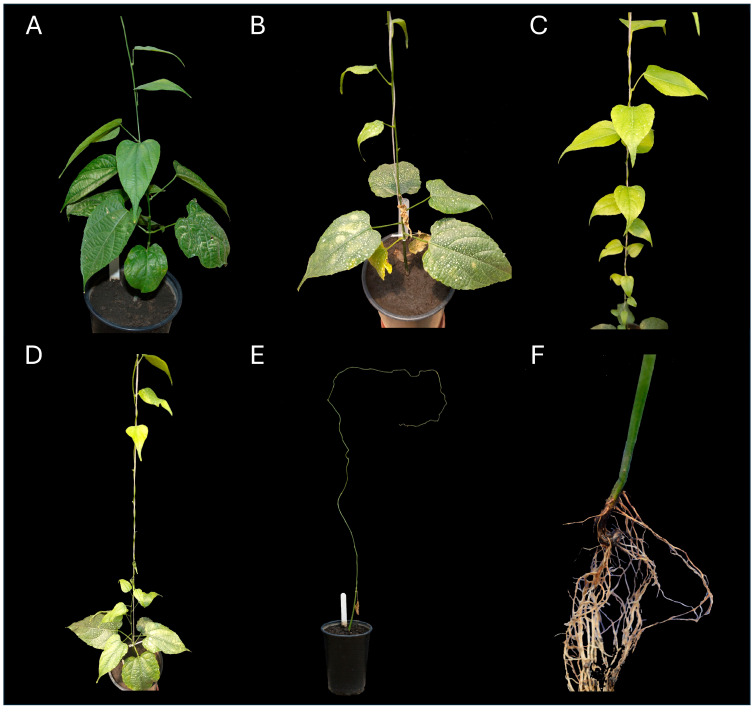
Symptoms in *P. volubilis* plants following inoculation with the *F. suttonianum* isolate: (**A**) healthy plants as control; (**B**–**D**) yellowing and leaf drop with *F. suttonianum* LBGM-FF01; (**E**,**F**) total defoliation of chlorotic leaves with root necrosis with *F. suttonianum* LBGM-FUSA001.

The pathogenicity of *Fusarium suttonianum* was confirmed of *P. volubilis* (sacha inchi) plants grown in pots under nursery conditions (Figure 5). Initial symptoms of the disease began to appear 20 days after artificial inoculation.

The plants inoculated with *F. suttonianum* exhibited an incidence rate of 100%, developing wilting symptoms similar to those observed in naturally infected plants. The control plants were asymptomatic. Fisher’s exact test revealed highly significant differences between the two groups (*p* < 0.001). The disease severity index was assessed approximately 106 days post-inoculation (dpi). The plants inoculated with *F. suttonianum* reached grade 3 on the scale (severe wilting and progressive defoliation of leaves towards the upper part), recording a severity index of 75%.

The pathogen was successfully re-isolated from the stem crown and roots from inoculated plants, showing identical morphological characteristics to the originally inoculated fungus. This result confirmed the fulfillment of Koch’s postulates, establishing *F. suttonianum* as the causal agent of the observed symptoms.

## 4. Discussion

This study reports, for the first time, root rot in *P. volubilis*, whose causal agent was identified as *F. suttonianum*. The identification was made through morphological (physical traits), molecular (*ITS* and *TEF-1α*) characterizations, as well as pathogenicity tests. *P. volubilis* is a crop of high nutritional value and an excellent source of high-quality vegetable oil, with considerable export potential for the San Martín region. Sacha inchi production represents a strategic opportunity to strengthen the regional economy, diversify exports, and promote sustainable development. However, knowledge of the diseases affecting this crop remains limited, underscoring the importance of identifying and characterizing the pathogens that compromise its yield and health.

*Plukenetia volubilis*, its cultivation is often constrained by both biotic and abiotic factors [71]. One significant limitation in sacha inchi production is its high susceptibility to nematodes. These parasites facilitate the entry of other pathogens, such as bacteria, fungi, and viruses, into the roots, posing considerable challenges for farmers [29,72]. Among fungal diseases, species of *Fusarium* are notable for causing root rot in this crop. Globally, various *Fusarium* species have been reported to cause root rot in *P. volubilis*. For instance, *F. solani*, *F. oxysporum*, *F. proliferatum*, and *F. graminearum* have been identified in China [26,27,28], while *F. solani* and *F. verticillioides* have been reported in Peru [29]. Guerrero-Abad et al. [29] also highlighted that *Fusarium* forms a complex with nematodes of the genus *Meloidogyne*.

In the San Martín region, the damage caused by *Fusarium* spp. in *P. volubilis* plantations has not yet been well documented. However, under nursery conditions, *Fusarium verticillioides* has been estimated to cause up to 55% seedling mortality [29]. Similar results were reported by Van et al. [73], who recorded root collar disease in sacha inchi seedlings in Vietnamese nurseries, with mortality rates ranging from 2.1% to 5.3%. In the field, the main phytosanitary problem affecting sacha inchi is represented by root-knot nematodes of the genus *Meloidogyne* spp., which, in combination with infections caused by *Fusarium* spp., induce severe biochemical and physiological alterations, including root decay, foliar chlorosis, and ultimately plant death [74].

In this context, our study presents the first report of *F. suttonianum* causing root rot in *P. volubilis* in the province of Picota, department of San Martín, Peru. *Fusarium* species are pathogenic to numerous agricultural crops, leading to significant economic losses. These fungi have a global distribution and are adept at surviving in diverse climates, causing a wide range of symptoms in various plant parts. They typically infect plants through wounds or natural openings [33,34,39,57].

*F. suttonianum* (syn. *Neocosmospora suttoniana*), also known as FSSC 20, belongs to the *Fusarium solani* species complex (FSSC) and is classified within Clade 3, the largest subgroup of the FSSC [59,75]. This fungus is recognized not only as a plant pathogen but also as an opportunistic pathogen affecting humans [64,76,77].

In our study, it was observed that in the species *P. volubilis*, F. suttonianum caused severe effects, including stunted growth, vascular wilt, and root rot. In plants, it has been identified as the causal agent of root rot in melon (*Cucumis melo*) in Brazil [64], where isolates of this species have caused symptoms similar to those observed in *Plukenetia volubilis*. The ability of *F. suttonianum* to adapt to diverse climates, coupled with its broad host range, underscores its significance as a threat to agriculture and public health. Its presence in plant families highlights its potential to cause substantial economic losses and its capacity to persist in the environment. For the management of root rot caused by *F. suttonianum*, the implementation of an integrated management plan is recommended [58,78].

The identification of *F. suttonianum* was accomplished using morphological characterization and phylogenetic analysis based on *ITS* and *TEF-1α* sequences. Morphological characteristics were determined by measuring the average size of macroconidia, microconidia, and chlamydospores. While morphological identification has been widely used in the past, it is now recognized that these features can vary significantly even within the same species [30,41,47]. The recorded morphological characteristics matched descriptions of *Fusarium suttonianum* [76], a member of the FSSC [79].

Currently, the molecular identification of *Fusarium* spp. often relies on genomic regions such as *TEF-1α*, β-tubulin (*βTUB*), calmodulin (*CAL*), the intergenic spacer (*IGS*), and RNA polymerase II subunits (*RPB1* and *RPB2*) [31,46,47,48,80]. However, sequencing one or two genes is now the standard for confirming the precise taxonomic characterization of species within the genus *Fusarium* [43].

*ITS* sequences were used, as they are highly effective for phylogenetic analyses of fungi, although not always reliable when identifying species based solely on a single gene [81,82]. *TEF-1α* was also used, as it is highly conserved and precise for identifying Fusarium species [30,51,80,81]. Phylogenetic analysis of *ITS* and *TEF-1α* sequences revealed that our strain LBGM-FF01 clustered within the FSSC, specifically *F. suttonianum*, aligning with morphological observations. This integrated approach of combining morphology and gene sequencing enhances the accuracy and reliability of *Fusarium* species identification, emphasizing the importance of multiple genetic markers for robust taxonomic characterization.

Pathogenicity tests confirmed that the field-isolated fungus produced the same symptoms in artificially inoculated plants. Furthermore, the study demonstrated that the severity of root rot in *P. volubilis* increases when root wounds are present, highlighting the critical role of the nematode–*Fusarium* complex in plant pathogenicity [29].

## 5. Conclusions

This study provides the first report of *Fusarium suttonianum* as the causal agent of root rot in *Plukenetia volubilis*. The pathogen’s identity was confirmed through morphological and molecular characterization. Molecular identification using *ITS* and *TEF-1α* sequences was crucial for providing a precise and accurate species identification. This discovery represents a critical first step in addressing root rot disease in sacha inchi, characterized by root decay and plant wilting. It serves as both a warning about the presence of this threat and a foundation for future research aimed at developing resistance to *F. suttonianum* in sacha inchi plants.

## Figures and Tables

**Table 1:** GenBank accession numbers of *Fusarium* spp. isolates used in this study for phylogenetic analysis.

Species	Isolate	GenBank Accession	
		*ITS*	*TEF-1α*
*F. suttonianum*	LBGM-FUSA001	PQ636870	PQ639345
*F. suttonianum*	NRRL:32858	DQ094617.1	DQ247163.1
*F. suttonianum*	NRRL:32316	DQ094413.1	DQ246944.1
*F. suttonianum*	145FUS	MW390928.1	MW389356.1
*F. suttonianum*	CGMHD4138	LC683311.1	LC683363.1
*F. suttonianum*	CGMHD2740	LC683304.1	LC683356.1
*F. suttonianum*	CGMHD2224	LC683301.1	LC683353.1
*F. suttonianum*	CGMHD0633	LC683273.1	LC683325.1
*F. suttonianum*	CGMHD1911	LC687548.1	LC697786.1
*F. suttonianum*	F27	PP421949.1	PP480018.1
*F. falciforme*	FSSC-Bi004P	KF647700.1	KF647715.1
*F. falciforme*	HN-01	PP779839.1	PP797138.1
*F. falciforme*	HN-02	PP779840.1	PP797139.1
*F. falciforme*	HN-03	PP779841.1	PP797140.1
*F. falciforme*	HN-04	PP779842.1	PP797141.1
*F. falciforme*	FSSC-N032P	KF647701.1	KF647716.1
*F. falciforme*	SP-Ff-Kd	PP851105.1	PP858878.1
*F. falciforme*	CBS475.67	NR_164424.1	LT906669.1
*F. solani*	LC13841	MW016727.1	MW620188.1
*F. solani*	LC13845	MW016731.1	MW620192.1
*F. solani*	LC13842	MW016728.1	MW620189.1
*F. solani*	LC13844	MW016730.1	
*F. solani*	GJS 09-1466	KT313633.1	KT313611.1
*F. solani*	NRRL:32484	DQ094449.1	DQ246982.1
*F. solani*	NRRL 43474	EF453097.1	EF452945.1
*F. solani*	LC13843	MW016729	MW620190
*F. keratoplasticum*	JMRC: NRZ:0164	MF467481.1	MF467460.1
*F. keratoplasticum*	JMRC: NRZ:0049	MF467482.1	MF467459.1
*F. keratoplasticum*	FRC S-2496	JN235276.1	JN235706.1
*F. keratoplasticum*	NRRL 32959	DQ094632.1	DQ247178.1
*F. keratoplasticum*	FSSCKer2	KX868663.1	KX266293.1
*F. keratoplasticum*	FSSCKer3	KX868664.1	KX266294.1
*F. oxysporum*	S11	MW019949.1	MT772142.1
*F. incarnatum*	LC13705	MW016532.1	MW594375.1
*F. equiseti*	NRRL20697	GQ505683.1	GQ505594.1

## Data Availability

The datasets used and analyzed in this study are available from the corresponding author upon reasonable request.
